# Breastfeeding initiation, duration, and experiences of mothers of late preterm twins: a mixed-methods study

**DOI:** 10.1186/s13006-022-00507-3

**Published:** 2022-09-08

**Authors:** Rakel B. Jonsdottir, Renée Flacking, Helga Jonsdottir

**Affiliations:** 1 Neonatal Intensive Care Unit, Landspitali – The National University Hospital of Iceland, Reykjavik, Iceland; 2 Faculty of Nursing, School of Health Sciences, University of Iceland, Reykjavik, Iceland; 3 School of Health and Welfare, Dalarna University, Falun, Sweden

**Keywords:** Twins, Late preterm, Breastfeeding, Mixed-methods

## Abstract

**Background:**

Twins and late preterm (LPT) infants are at an increased risk of being breastfed to a lesser extent than term singletons. This study aimed to describe the initiation and duration of any and exclusive breastfeeding at the breast for mothers of LPT twins and term twins during the first 4 months and to explore the breastfeeding experiences of mothers of LPT twins.

**Methods:**

A sequential two-sample quantitative–qualitative explanatory mixed-methods design was used. The quantitative data were derived from a longitudinal cohort study in which 22 mothers of LPT twins and 41 mothers of term twins answered questionnaires at one and four months after birth (2015–2017). The qualitative data were obtained from semi-structured interviews with 14 mothers of LPT twins (2020–2021), based on results from the quantitative study and literature. Analysis included descriptive statistics of quantitative data and deductive content analysis of the qualitative data, followed by condensation and synthesis.

**Results:**

All mothers of LPT twins (100%) and most mothers of term twins (96%) initiated breastfeeding. There was no difference in any breastfeeding during the first week at home (98% versus 95%) and at 1 month (88% versus 85%). However, at 4 months, the difference was significant (44% versus 75%). The qualitative data highlighted that mothers of LPT twins experienced breastfeeding as complex and strenuous. Key factors influencing mothers’ experiences and decisions were their infants’ immature breastfeeding behaviors requiring them to express breast milk alongside breastfeeding, the burden of following task-oriented feeding regimes, and the lack of guidance from healthcare professionals. As a result, mothers started to question the worth of their breastfeeding efforts, leading to changes in breastfeeding management with diverse results. Support from fathers and grandparents positively influenced sustained breastfeeding.

**Conclusions:**

Mothers of LPT twins want to breastfeed, but they face many challenges in breastfeeding during the first month, leading to more LPT twins’ mothers than term twins’ mothers ceasing breastfeeding during the following months. To promote and safeguard breastfeeding in this vulnerable group, care must be differentiated from routine term infant services, and healthcare professionals need to receive proper education and training.

**Supplementary Information:**

The online version contains supplementary material available at 10.1186/s13006-022-00507-3.

## Background

Globally 1.5 to 3% of live births are twin births [1] and twins are considered to be a group that is at a higher risk for complications perinatally and long-term [2, 3]. The risk of prematurity is much higher for twins than for singletons: 63% of twins are born preterm (< 37 weeks’ gestation) compared to about 5–8% of singletons. Out of those twins born preterm, 41% are born late preterm (LPT), i.e., between 34^0 / 7^ to 36^6 / 7^ weeks [4, 5].

Breast milk is universally acknowledged as the optimal source of nutrition for infants. Breast milk contains not only important nutrient factors but also non-nutrient factors, including immunoglobulins and lactoferrin, that may promote intestinal adaptation and maturation, improve enteral feeding tolerance, and protect against infective and inflammatory diseases [6, 7]. Even so, and despite the increased knowledge of the benefits of breast milk, many preterm infants and twins are not breastfed or do not get any breast milk during the first months of life. Breastfeeding rates for term and preterm twins are lower than those for term and preterm singletons; mothers of twins have a lower breastfeeding initiation rate than mothers of singletons and wean breastfeeding earlier than mothers of singletons [8–13]. The breastfeeding initiation rate for twins ranges from 38 to 80%, and the rate of any breastfeeding at 6 months from 10 to 50%, with 8–22% of twins breastfeeding exclusively [8, 9, 12–17]. These large variations in breastfeeding rates may depend on culture and access to healthcare services, on how breastfeeding has been defined, and differences within study populations, such as inclusion criteria.

The risk for ceasing breastfeeding in mothers of twins, as well as in mothers of LPT infants, is higher in mothers who are ill or fatigued, who smoke, lack support, lack breastfeeding experience, have a low milk supply, and in those whose infants are preterm or ill at birth [9, 17–21]. In mothers of infants who are born twins and LPT, the associated factors on breastfeeding outcomes are not well understood. To our knowledge, no study has been published focusing specifically on breastfeeding in LPT twins. However, some studies have included smaller subgroups of LPT twins. Crippa et al., [22] concluded that having twins negatively influenced breastfeeding of late preterm infants, and Gianni et al., [23] showed that mothers of LPT infants considered having twins as a barrier to breastfeeding. Several studies note that although the same challenges may influence mothers of twins and mothers of singletons, mothers of twins are faced with unique circumstances to maintain breastfeeding [17, 20, 24]. The same is true for mothers of LPT singletons compared to term singletons [22, 25]. Thus, if twins are born LPT, it adds to the challenges. Breastfeeding is a complex psychosocial and biological process, especially in the case of twins, and is mutually influenced by mother and infants. Explorations of maternal experiences are needed to design interventions with practical relevance and improve LPT twins’ breastfeeding outcomes. Thus, this study aimed to describe the initiation and duration of any and exclusive breastfeeding at the breast for mothers of LPT twins and term twins during the first 4 months and to explore the breastfeeding experiences of mothers of LPT twins.

## Methods

### Study design

This was a two-phase sequential explanatory mixed-method study [26]. Sequential explanatory study design is characterized by two distinct phases: a quantitative data collection and analysis phase, followed by a qualitative data collection and analysis phase. The aim of the qualitative phase was to help explain and elaborate on the results obtained in the first phase [26, 27]. According to this mixed-method study design, the first phase consisted of an analysis of the quantitative data on all twins in a longitudinal cohort survey of mothers of LPT (34^0 / 7^ to 36^6 / 7^ weeks) infants and term (37^0 / 7^ to 41^6 / 7^ weeks) infants [28, 29]. In the second phase, the qualitative phase, issues from the first phase and literature were explored in individual semi-structured interviews with another population of mothers of LPT twins. The issues identified in the first phase of the study together with key issues of the literature were included in the schedule for the individual semi-structured interviews which also comprised a predetermined framework – a categorization matrix – that was used for the data analysis [26, 30]. Data from both quantitative and qualitative phases were triangulated according to the aims of the study.

### Setting and context

Both phases of the study were conducted at The National University Hospital of Iceland (NUH), which is the country’s largest birth facility, with about 3500 births per year. It is the main referral hospital for high-risk pregnancies and childbirths in Iceland, where 75% of all deliveries and about 95% of preterm (< 37 weeks) deliveries occur. The setting of the study and the healthcare context is further described in Supplemental Table 1.

### Participants and data collection

#### The quantitative phase

##### Participants

The mothers of twins participating in a longitudinal cohort study between mid-March 2015 and the end of May 2017 were included [28, 29]. The exclusion criteria were as follows: newborns with a major congenital abnormality; newborns with an Apgar score of ≤6 at 5 min or a clinical definition of injury to the central nervous system; maternal age < 18 years; and mothers who were unable to speak and read Icelandic.

During the research period, 132 mothers gave birth to twins at the NUH; 70 delivered at term, and 62 gave birth to preterm twins, of which 43 mothers gave birth to LPT twins. In total, 63 mothers participated; 22 were mothers of LPT twins, and 41 were mothers of term twins, which rendered a participation rate of 65%. Forty-eight twin s’ mothers (76%) answered at both study times (17 LPT twin s’ mothers and 31 term twins’ mothers); 15 mothers either answered at 1 month or at 4 months. Out of the 34 mothers who were invited to participate but did not, nine were unreachable (phone and address), six accepted but did not answer the questionnaires, and the remaining 19 mothers declined participation.

##### Recruitment and data collection

Every 2 weeks, a list of all twin births at the NUH was reviewed, and mothers that met the inclusion criteria were invited to participate by mailed letters, in which they were asked to respond to an email if they wanted to participate. If mothers did not answer by email, a research assistant phoned them and invited them to participate. The mothers were informed, both on the phone, in the mailed letter, and in the email, that participation in the study was voluntary and that they could withdraw at any time. Answering the electronic questionnaire was considered to represent informed consent. The mothers that agreed to participate by answering the email or agreeing during the phone call were registered in a Research Electronic Data Capture (REDCap) (Vanderbilt University, Tennessee, USA) database [31]. Two weeks later, the software automatically sent out an email with the questionnaire, around the time when the infant was 1 month of postnatal age and again at 4 months of postnatal age. If the questionnaire was not filled out within 5 days of receiving the email, the software automatically sent out a reminder and again after another 5 days if still no response had been recorded. REDCap electronic data capture tools were hosted at the University of Iceland.

#### The qualitative phase

##### Participants

Mothers of LPT twins giving birth from the beginning of June 2020 to the end of March 2021 at the NUH were invited to participate in the qualitative phase of the study. Mothers who did not speak Icelandic or whose infant(s) had died after birth were excluded. During the research period, 24 mothers gave birth to LPT twins, and 21 were eligible for participation (three did not speak Icelandic). There were 15 mothers who accepted the invitation, of whom 14 participated, which rendered a participation rate of 67%.

##### Recruitment and data collection

Every 2 weeks, from the 1 June 2020 to 31 March 2021, a list of all mothers giving birth to twins at the NUH was reviewed. Mothers who met the inclusion criteria were invited to participate by a mailed letter in which they were asked to respond to an email or by phone if they wanted to participate in semi-structured individual interviews when their infants were four to six months of postnatal age. If mothers did not answer, they received a phone call inviting them to participate. The mothers were informed, on the phone, in the mailed letter, and in the email. Participation was voluntary; the mothers were informed that they could withdraw at any time. Written consent was obtained.

### Measures

#### Quantitative phase

Data on demographics for the infants and mothers were obtained from electronic medical and hospital records. An electronic survey with questions on feeding and maternal well-being was sent to the mothers when their infants were one and four months of postnatal age. Feeding questions covered what the infants were fed, the feeding method and frequency, the infants’ behaviors, and potential difficulties. Breastfeeding was defined as feeding at the breast and exclusive breastfeeding when a mother fed only breast milk directly from the breast but could include medications and vitamins [32]. We also investigated breast milk feeding and categorized it as: exclusive (i.e., only breast milk but could include medications, fortification, and vitamins); partial breast milk (breast milk in combination with infant formula); and no breast milk (infant formula feeding with no breast milk intake), all regardless of method [33].

Maternal well-being comprised questions on anxiety and depression 12 months before birth. The mothers were asked about their level of worries related to the infants’ nutrition, health, sleep, crying, weight, and development at one and four months of infants’ age. Worries were defined as concerns about those factors. In addition, depressive symptoms were measured by the Edinburgh Postnatal Depression Scale (EPDS) at one and four months. The scale includes ten items, with a total score of 0–30, and a higher score indicates more depressive symptoms. A cutoff point of ≥13 was used as recommended by Cox et al., [34]. Fewer than 5% of the data were missing for all variables. A more detailed description of the measures has been presented in our papers on LPT singletons [28, 29].

#### Qualitative phase

The semi-structured interviews were conducted between mid-December 2020 and the end of August 2021. The first author, an experienced neonatal nurse and Ph.D. candidate, conducted the interviews. Twelve face-to-face interviews were conducted in the mothers’ homes, and two video-phone interviews were conducted, as per the mothers’ requests, making in total 14 interviews. The audio-recorded interviews lasted 40–100 minutes, with an average length of 57 minutes. A semi-structured interview guide was developed based on key findings from the quantitative phase and the literature, including factors influencing the feedings of LPT twins during the first 6 months, with particular attention to the perceptions of and attitudes toward breastfeeding, obstacles, and helpful factors.

The first author worked in the NUH NICU during the interviews but had no personal contact with the participants. RF and HJ, professors in nursing with substantial qualitative research experience, guided the design, process, and analysis of the interviews.

### Data analysis

**Fig. 1:**
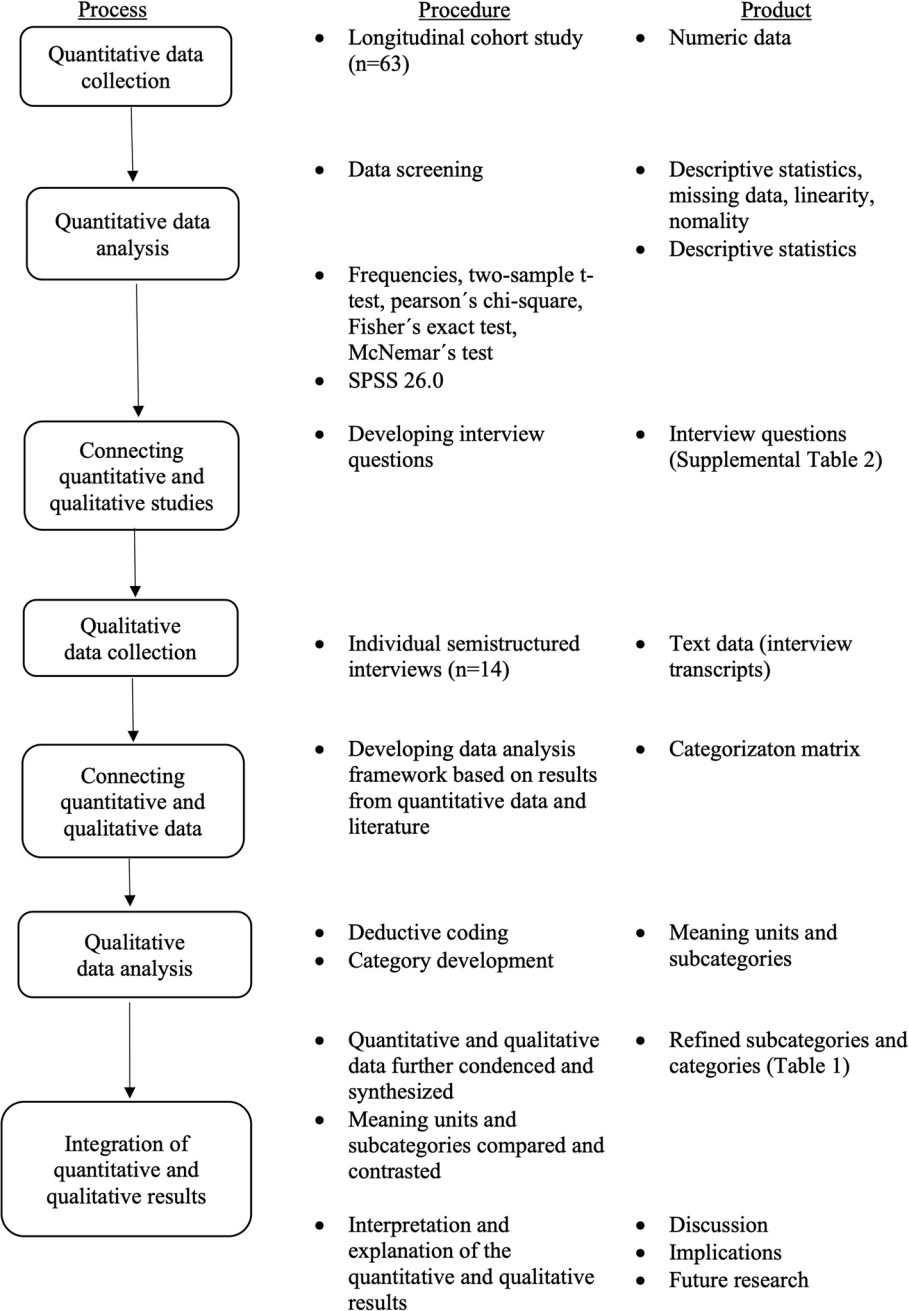
Visual model of the mixed-method sequential explanatory research process

Data were analyzed and synthesized in three steps informed by an approach recommended by Ivankova and colleagues [26]. See Fig. 1.

#### Step 1 (quantitative phase)

The data were analyzed with descriptive statistics by calculating means with standard deviations, and medians with interquartile ranges or percentages, according to the type and distribution of the data. The two twin groups, LPT and term-born infants, were compared by two-sample *t*-tests, Pearson’s Chi-square tests, or Fisher’s exact test, when appropriate, with a two-sided, 5% level of significance. McNemar’s test was used to assess the differences in the proportions of breastfeeding at the breast from the first week at home to 4 months within the groups. A *P*-value of less than 0.05 was considered statistically significant. Statistical analyses were performed using SPSS software, version 26.0 (IBM Corp, New York, USA). Some mothers (< 10%) answered the questionnaire only for one twin, primarily for twin A. In these cases, the answers were duplicated for the missing twin.

#### Step 2 (qualitative phase)

First, a categorization matrix including items from the quantitative study (step 1) and the literature was constructed [26]. A research assistant fully transcribed the qualitative data from the face-to-face interviews. The first author (RBJ) thereafter read the transcripts while listening to the recordings of the interviews to ensure accuracy and correct errors. Then two researchers (RBJ, HJ) independently reviewed the transcripts and analyzed the data by means of deductive qualitative content analysis, based on the previously determined categorization matrix [30]. The interview transcripts and the categorization matrix were compared and contrasted. Items / categories relevant to the categorization matrix were highlighted, then extracted, and placed in potential subcategories. Then the items / categories were compared, contrasted, and rearranged into subcategories and categories by discussions among all authors (RBJ, HJ, RF). Independent analysis and conversations between the two authors (RBJ, HJ), as well as discussions among all authors (RBJ, HJ, RF), increased the credibility of the findings [30].

#### Step 3 (data synthesis)

The data analyzed in Steps 1 and 2 were condensed and synthesized (integrated) into subcategories and categories. The content of each of the subcategories was compared and contrasted to keep the subcategories and categories as mutually exclusive as possible.

### Ethical aspects

The National Bioethics Committee of Iceland, the country’s data protection authority, and the Medical Director of The National University Hospital of Iceland (14–051-V1, 2014030541AT, 16 LSH 45–14) approved the longitudinal cohort study. The Institutional Review Board (LSH 38–20) and the Institutional Research Committee (vrn 16–20) of the National University Hospital of Iceland approved the qualitative study.

## Results

### Participant characteristics

The characteristics of the LPT twins and their mothers in the longitudinal cohort study (hereafter referred to as the quantitative phase) and in the interview study (hereafter referred to as the qualitative phase) are presented in Table 1. The LPT twin groups were comparable in both studies; apart from that, more multiparas and mothers of boys were in the qualitative sample.

**Table 1:** Characteristics of the participating mother and twins in the quantitative study (63 / 126) and qualitative study (14 / 28)

	Quantitative					Qualitative	
	LPT		Term		***p***	LPT	
	n	%	n	%		n	%
**Mother**	***n*** **= 22**		***n*** **= 41**			***n*** **= 14**	
Vaginal birth	16	73%	35	85%	0.253	9	62%
Married / living with partner	20	95%	36	95%	> 0.05	14	100%
Maternal age, mean (SD)	29.91 (5.06)		32.29 (5.99)		> 0.05	30.8 (3.12)	
Household income / month (ISK)					> 0.05		
< 400.000	6	28%	5	13%			
4–800.000	10	48%	18	49%			
> 800.000	5	24%	14	38%			
Highest educational level					> 0.05		
University	11	55%	30	79%		10	71%
High school	7	35%	5	13%		4	29%
Elementary	2	10%	2	8%		0	0%
Illness last year							
Depression	3	14%	4	10%	0.637	0	0%
Anxiety	3	14%	4	10%	0.637	2	14%
Well-being after birth							
EPDS at 1 month, mean (SD)	6.5 (3.19)		4.73 (3.06)		**< 0.05**		
EPDS at 4 months, mean (SD)	5.29 (3.10)		4.31 (3.44)		> 0.05		
Multipara	10	45%	26	65%	> 0.05	11	79%
Have breastfed before	10	100%	24	92%	> 0.05	11	100%
**Infant**	***n*** **= 44**		***n*** **= 82**			***n*** **= 28**	
NICU	21	48%	0	0	**< 0.001**	16	57%
Hospital stay							
< 24 hours	0	0	4	5%	0.133	0	0%
1 to 6 days	25	57%	72	89%	**< 0.001**	14	50%
7 to 13 days	17	39%	2	2%	**< 0.001**	14	50%
14 to 28 days	0	0	2	2%	0.347	0	0%
> 29 days	0	0	0	0	> 0.05	0	0%
Gestational age weeks							
34 + 0–34 + 6	10	23%				6	21%
35 + 0–35 + 6	12	27%				10	36%
36 + 0–36 + 6	22	50%				12	43%
37 + 0–37 + 6			68	83%			
38 + 0–38 + 6			12	15%			
39 + 0–39 + 6			2	2%			
≥ 40			0	0%			
Breastfeeding							
First breastfeeding	n	%	n	%		n	%
< 6 hours	22	52	71	89	**< 0.001**	11	39
6–24 hours	13	31	6	7	**< 0.001**	7	25
> 24 hours	7	17	0	0	**< 0.001**	10	36
Did not breastfeed at the hospital	0	0	3	4	0.181	0	0
First pumping							
< 6 hours	4	19	4	10	0.317		
6–24 hours	16	76	13	32	**0.001**		
> 24 hours	1	5	12	30	**0.022**		
Did not pump at the hospital	0	0	11	28	**0.006**		
Gestational age at birth in days, mean (SD)	250.45 (5.78)		262.85 (3.17)		**< 0.001**	248.86 (5.22)	
Birth weight, grams, mean (SD)	2483.32 (338.78)		2835.04 (364.33)		**< 0.001**	2388.79 (319.0)	
Apgar 5 min, median (SD)	9.25 (0.87)		9.45 (0.74)		> 0.05		
Girl	21	48%	35	43%	> 0.05	8	29%
Weight at 1 month, grams, mean (SD)	3365.83 (716.01)		3746.43 (788.24)		**< 0.05**		
Weight at 4 months, grams, mean (SD)	5665.76 (965.48)		5786.58 (1389.32)		> 0.05		
Age when answering at time 1, days, (SD) (range)	42.43 (7.70) (27–60)		44.31 (11.48) (25–77)		> 0.05		
Age when answering at time 2, days, (SD) (range)	133.24 (9.44) (117–156)		136.46 (13.48) 120–173		> 0.05	160 (14.63) (138–189)	

*ISK* Icelandic krona, *LPT* late preterm, *SD* standard deviation

### Breastfeeding rates

**Fig. 2:**
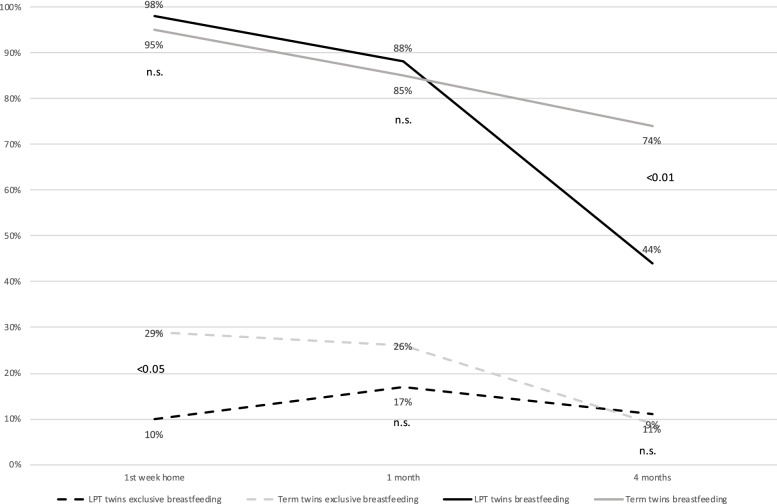
Proportion of any breastfeeding at the breast and exclusive breastfeeding at the breast in term twins (*n* = 80) and late preterm twins (*n* = 42)

The breastfeeding rates (any and exclusive) in mothers of LPT and term twins are presented in Fig. 2 and Table 2.

**Table 2:** Comparison of breastfeeding progression, interventions, and behavior of late preterm twins (*n* = 44) and term twins (*n* = 82)

	LPT twins						Term twins								
	1st week		1 month		4 months		1st week		1 month		4 months				
	n	%	n	%	n	%	n	%	n	%	n	%	*P* - value	*P* - value	*P* - value
Insufficient milk supply	11	52	9	43	8	38	19	48	15	38	14	35	0.764	0.701	0.815
Abundance of milk	4	19	4	19	2	10	9	23	2	5	3	8	0.715	0.078	0.789
Breast milk feeding															
Exclusive	6	14	10	24	4	11	23	29	26	33	6	9	0.060	0.295	0.304
Partial	36	86	28	67	12	33	53	66	44	55	42	66	**0.016**	0.193	**< 0.001**
No	0	0	4	9	20	56	4	5	10	12	16	25	0.133	0.609	**< 0.001**
Methods for feeding (several methods optional)															
Breast	41	98	37	88	16	44	76	95	68	85	48	75	0.412	0.645	**< 0.001**
Bottle	38	90	35	83	31	86	57	71	58	73	49	77	**0.015**	0.209	0.229
Finger feeding	6	14	0	0			17	21	3	4			0.337	0.181	
Cup feeding	4	10	0	0			13	16	2	3			0.355	0.248	
Supplemental nursing system	6	14	1	2			4	5	2	3			0.079	0.740	
Feeding tube	3	7	0	0			0	0	0	0			**0.016**	> 0.05	
Test weighing	24	57	7	17			36	45	6	8			0.201	0.127	
Used a nipple shield	14	33	12	29	0	0	11	14	6	8	0	0	**0.012**	**0.002**	> 0.05
Pacifier use	29	67	39	93			34	43	68	85			**0.011**	0.193	
Weak suck	24	57	13	31			24	30	10	13			**0.003**	**0.015**	
Little stamina	36	86	19	45			44	55	21	26			**< 0.001**	**0.031**	
Sleepy infant need to wake to drink	27	64	10	24			39	49	16	20			0.109	0.603	
Started solids					25	57					34	53			0.669

*LPT* late preterm

The analysis of the LPT twin s’ mothers’ experiences and the synthezation of the quantitative and qualitative data, resulted in two main categories and nine subcategories, which describe and illuminate the influencing factors on LPT twins’ breastfeeding progression (Table 3).

**Table 3:** The categories and subcategories

Categories	Subcategories
The first month: a complex and strenuous phase where task-oriented feeding regimes were followed	• Initiation • Immature breastfeeding behaviors • Mothers’ health • Milk expression • Focus on weight: scheduled feedings, set amounts of milk, and test-weighing • Transition from hospital to home
Months two to four and navigation through feeding: finding your own path	• A constant struggle and tiredness with an uncertain outcome • Managing life and feeding: questioning the worth of breastfeeding efforts • Need for external support

### The first month – a complex and strenuous phase where task-oriented feeding regimes were followed

The quantitative and qualitative findings on the LPT twins’ mothers’ experiences during the first month are presented in six subcategories, which describe influencing factors on breastfeeding.

#### Initiation

The quantitative data showed that the breastfeeding initiation rate was high in both twin groups, as 100% of the LPT twins and 96% of the term twins were breastfed at the hospital after birth (*p* >  0.05). The first attempt at breastfeeding within the first 6 h after birth occurred for 52% of the LPT twins versus 89% of the term twins (*p* <  0.001). All mothers in the qualitative study had the intention before birth to breastfeed, but many of them experienced that their infants’ or their own health and / or mother / infant separation hindered breastfeeding. A mother who had a cesarean section said:

*“They were born, and [infant’s name], the smaller one, he was sort of taken right away and put into the incubator, but [other twin’s name], he got to stay for a short time with us. But then he was also taken to the incubator in the NICU. [Dad’s name] went with them, and I was there [in the delivery suite] to recover.”* (M4)

#### Immature breastfeeding behaviors

Significantly more mothers of LPT twins than term twins reported a weak suck during the first week at home and when the infants were at 1 month of age. Little stamina while breastfeeding was more often reported by mothers of LPT twins than mothers of term twins, both during the first week home and at 1 month of age. Significantly more mothers of LPT twins also reported that they always needed to wake their infants for breastfeeding during the first week at home (Table 2).

In the interviews, mothers highlighted that the LPT infants’ weak suck, little stamina, and sleepiness affected breastfeeding progression considerably during the first month. The mothers described their twins as not interested in feeding, always falling asleep at the breast, always needing to be woken up, and not sucking at all or sucking poorly when put to the breast. As one mother noted, *“You know they sucked a little and then just went to sleep; they were so tired.” (M6)*. Thus, because of the infants’ immature breastfeeding behavior, breastfeeding and feeding were regarded as strenuous and took considerable time.

#### Mothers’ health

Some mothers experienced anxiety and / or depression during the year before birth. At 1 month, only one mother of twins in the LPT group and no mothers in the term group had an EPDS score of ≥ 13. However, the mean EPDS score of the LPT twins’ mothers at 1 month was significantly higher than the mean score of the term twins’ mothers (Table 1).

Mothers experienced that their health problems affected breastfeeding; some had complications lasting for weeks or months after a cesarean section, whereas others recovered quickly. Pregnancy-related morbidity, like preeclampsia and hyperemesis, was also relatively frequent. A mother with severe preeclampsia explained why she did not breastfeed her twins much during the first days after birth: *“I was in bed, and I had a catheter and could not walk or talk. I was sort of out of it for about two days after birth, and I could not see my children,”* (M2). Fatigue and exhaustion during the first days and weeks after birth was also common and affected breastfeeding:

*“I had some kind of severe exhaustion. It came after a few days, perhaps on day four or five, and I thought I was getting sick. I had flu-like symptoms every day. It happened late in the day, about five o’clock, and I just fell asleep and could not control it. I just fell asleep, and [husband’s name] had trouble waking me up to breastfeed. This just happened over and over again.”* (M4).

#### Milk expression

The use of a breast pump for expressing breast milk and establishing milk production was significantly more common among mothers of LPT twins compared to mothers of term twins (Table 1). All mothers of LPT twins expressed breast milk after birth, but the time of initiation, frequency, and effectiveness varied. Some had their breasts hand expressed shortly after birth: *“She [the midwife] came right away and worked hard on me to get the colostrum out to give to them [her twins]” (M3)*. All mothers used the breast pump to establish milk production alongside breastfeeding at the breast, with the goal to pump for a short time and to only breastfeed at the breast later on, as described by a mother:

*“After every feeding for many weeks after birth, I used the breast pump to get more milk. The plan was always to breastfeed exclusively. Even though deep down, I knew it was highly unlikely that I could do that. But I thought, let’s try it.”* (M1).

Not having enough breast milk was common for all the twins’ mothers, with no difference between the mothers of LPT and term infants (Table 2). Some of the interviewed mothers experienced an insufficient milk supply only during the first week after birth, as described by a mother who pumped every 3 h after birth: *“.*. *. I was pumping full time. .. always. .. I was pumping for every feeding but had to give extra. .. They got perhaps 40% formula; the rest was breast milk.” (M3)* Many mothers continued to struggle with milk production and breastfeeding and supplemented with infant formula:

*“Breastfeeding twin B was a struggle. I never knew if [infant’s name] would take the breast. But both of them got better, and I thought we were almost there [breastfeeding exclusively]. So yeah, but it never happened. I did not have enough milk.”* (M10)

The burden of insufficient milk production and / or having to use a breast pump at the same time as focusing on establishing breastfeeding were challenging for the mothers and impacted their experiences and actions:

*“We always hoped for exclusive breastfeeding, but it never happened. .. perhaps partly because I had such limited time to use the breast pump. .. I got so confused in all this [breastfeeding at the breast and expressing] that sometimes I just forgot why I was doing all this.”* (M1)

#### Focus on weight-scheduled feedings, set amounts of milk, and test-weighing

The mothers experienced major concerns for their LPT twins’ weight gain during the first weeks. These concerns motivated the mothers to use numerous feeding regimes related to the infants’ breast milk intake and breastfeeding (Table 2). The timing of breastfeeding was predetermined by scheduled feeding times, the amount of milk in a feed was prescribed as a fixed number of milliliters, and test weighing was used as a method to determine the infant’s milk intake. As a consequence, breastfeeding became “robotic” and “instrumental” during the first month, where the mothers used the words *“military camp”* and *“boot camp”* to describe the experience. The mothers would start breastfeeding at a set time, and then the mother, or partner, would bottle feed afterward with a fixed amount of milk. Thus, feeding became a task: “*We were just working on getting them to gain weight. .. That was what life was all about. .. Yes, yes, this was a task.”* (M1)*.* The mothers did not talk about the infants’ cues but talked much about the standardized schedules assigned by health professionals and ways to ensure that their infants got enough milk during feeds.

*“I was so stressed about this [weight gain]. I focused so much on making sure, and therefore I was never careless about this [breastfeeding]. The schedule was three hours, and even if I was tired during the nights, I just thought, ‘no, I must get out of bed,’ and they [her twins] must do it [breastfeed]. Although they were lazy, I never gave up, and I was like, ‘You are not finished!’ This is what it was like, but when I saw that they had gained weight—they gained a lot—the weight curve was just straight to the ceiling, and I became calmer.”* (M4)

Further, test-weighing was commonly used by the mothers to assess the intake of breast milk (Table 2). One mother who test-weighed her LPT twins for 6 weeks described her experiences:

*“We rented a scale that we placed on this table and weighed them. We wrote down how much they weighed and, of course, calculated how much they needed in addition to breastfeeding. After four days, we felt this was insane.”* (M1).

In addition, nipple shields were used to “facilitate” the transfer of milk and to establish breastfeeding at a faster speed. Significantly more mothers of LPT twins indicated that they used a nipple shield during the first week at home and at 1 month compared to mothers of term twins (Table 2).

#### Transition from hospital to home

During the transition from hospital to home, mothers experienced lack of support and guidance from healthcare professionals. A majority of the mothers of LPT twins (62%) and term twins (55%) had sought help and guidance when the infants were 1 month old, mostly from healthcare staff and friends / family but also on the internet. There were more mothers of LPT twins than mothers of term twins that did not get the help and support when needed, 23% versus 10% respectively, although this was not statistically significant (not presented in a table).

The LPT twins’ mothers’ experiences of support and guidance in the NICU and the maternity unit were noticeably different. The mothers with twins in the NICU viewed the transition to home positively. They felt prepared and experienced the feeding of the twins as less complicated than the mothers with twins in the maternity unit did. A mother from the NICU described her experiences:

*“I was so glad for the NICU stay. Tube feeding the twins gave me time to adjust. I was still trying to get the hang of breastfeeding. If the tube feeding had ended earlier, I would not have succeeded in breastfeeding them.”* (M5)

In contrast, LPT twins’ mothers with twins in the maternity unit felt insecure and unprepared; they felt that feeding their infants was complicated and did not feel prepared when they went home. They lacked guidance on how to proceed with breastfeeding, both from staff in the maternity unit, and the home midwives. They, as well, lacked information, e.g., on the effects of prematurity on infants’ breastfeeding behaviors, milk production, and the importance of using a breast pump. A mother who stayed in the maternity unit for 2 days, which was the standard practice, thought upon discharge, that everything was going fine, but did not realize that her twins, were not feeding properly:

*“Perhaps if I had pumped more and received proper information during the stay in the maternity unit, I would have better understood why and how often I should be pumping because I had not needed to pump while breastfeeding my older child. I needed guidance about why I had to pump so frequently and its implications for milk production and breastfeeding. Because I thought that they [the twins] were doing a good job at the breast, and therefore my milk production would be fine. However, they were, of course, not strong enough.” [The mother stopped breastfeeding during the second month because of perceived too little milk production, although she truly wanted to breastfeed]* (M10)

Another mother who breastfed and topped up with finger-feedings, as per recommendations from the maternity unit, was overwhelmed during the second week at home because of the complexity of the feedings: *“At that time, I was so overwhelmed. It was so much and so complicated that I almost could not bear to go out of bed.”* (M12).

The mothers of LPT twins highly regarded the home midwives and considered them “experts” on health after birth, breastfeeding, and feeding strategies. However, although the home midwives were an important resource for support, several mothers indicated that their guidance was sometimes conflicting and damaging. One mother stated: *“I felt like she [the home midwife] considered me to know more than I did about breastfeeding; ‘You have had a baby; this is not your first one.’.*. *.”* (M6). Another mother who struggled with milk production experienced that guidance from the home midwife might have negatively influenced her breastfeeding:

*“She [the home midwife] told me not to pump during nights, which I did. But in retrospect, considering things that I have heard [during the last months], I think that this might have been a mistake, as I just never had enough milk.”* (M10)

All mothers, regardless of the unit where they stayed after birth and the length of hospital stay, had home visits from a primary healthcare nurse throughout the first month, followed by baby check-ups in the primary healthcare center in the following months. There were mixed feelings about that service. The mothers did not consider the primary care nurses to be knowledgeable in breastfeeding preterm infants*: “I thought somehow that their guidance would include feeding strategies after discharge and especially with preterm infants. .. No one talked to me about that, and that was not good.”* (M5).

### Months two to four and the navigation through feeding: finding your own path

The quantitative and qualitative findings on the LPT twin s’ mothers’ experiences during months two to four are presented in three subcategories.

#### A constant struggle and tiredness with an uncertain outcome

The LPT twin s’ mothers described the time between two and four months as hectic and crazy, with sleepless nights; the days and nights were filled with tasks and too few hours to do everything that needed to be done. They used phrases like” *it is all in a mist,”* “*it is all blurred,” “I did not sleep,” “I was so tired,”* and*” it has been the most difficult time in my life*.” A lack of sleep and worries affected many mothers physically and mentally and influenced breastfeeding considerably.

*“I calculated that I was breastfeeding for 8 out of 24 hours. It is, of course, a full-time job. People have difficulties sitting for 8 hours. When you have done so for one, two, or three months, then your body is like. .. you feel it. .. That is what I experienced somehow; you are struggling with breastfeeding but somehow not doing enough.”* (M1)

Struggles with milk production were still common during this time; some also struggled with breastfeeding at the breast, while for others, breastfeeding was improving despite perceived too little milk production. Hence, feeding was complicated, milk production was uncertain, the result of their breastfeeding struggles was uncertain, and they were tired, which influenced the decisions on breastfeeding, as described by a mother:*“I used the breast pump every time I breastfed for six weeks so that I would have breastmilk to give them [the twins] in the bottle when they did not finish their feedings at the breast. Sometimes, although I had to use formula, and at that time, I simply had had enough, and they [the twins] were starting to take the breast fine. So, I decided that I would only use formula in the bottle, quit pumping, and what happened would happen.”* (M3) Another mother of LPT twins described that she chose to focus less on milk production, even though she knew the outcome for breastfeeding might be negative:

*“I had been doing this [pumping after every breastfeeding] for several days, just not doing anything other than breastfeeding and pumping, having even less sleep at night than before. Then, I thought, ‘This is starting to be a burden,’ it cannot overshadow the time spent with the family and other things I want to do. So, I quit pumping and started to use formula.”* (M12)

#### Managing life and feeding: questioning the worth of breastfeeding efforts

Most of the mothers experienced that the LPT twins’ breastfeeding behaviors were getting better around the time they reached full term. However, as very few mothers exclusively breastfed at the breast at this time, breastfeeding did not become more manageable during the following months. The burden of the complex management of the twins’ feedings (e.g., breastfeeding, pumping, bottle feeding), in addition to perceived insufficient milk production, peaked when the infants were one to three months old and were the key factor in mothers’ breastfeeding decisions during this time. The search for appropriate breastfeeding management, or a feasible routine that allowed breastfeeding exclusively or in combination with bottle feeding, preferably without expressing, was also evident. Some mothers succeeded in finding a breastfeeding routine that was not burdensome and suited their life, and enabled them to do things other than just breastfeed and pump. Others did not; they experienced breastfeeding as too burdensome, felt that their breastmilk production was too little, and that their efforts were therefore not worth it, and consequently ceased breastfeeding: *“I just got sick of this always being on the clock. .. And just somehow always experienced like it was not enough. .. I wanted to live life and enjoy it.”* (M1). In the mothers’ search for a manageable breastfeeding routine, some simplified feeding by stopping expressing breast milk. One mother that ceased breastfeeding when her twins were 3 months of age stated:

*“During the second month, I quit pumping. I just had enough and found it easier to just breast- and bottle feed. Pumping interrupted my sleep at night and just everything. We had three weeks where it was going fine, but suddenly, there was no milk.”* (M1)

Other mothers started to bottle feed so that other people could help: “*We started to use the bottle so that he [the father] could participate in the feedings and perhaps so that I could have some breaks.”* (M14). By quitting expressing and / or breastfeeding less often, milk production often decreased, and / or the benefits of the bottle feeding gradually outweighed the benefits of breastfeeding, and subsequently, many mothers ceased breastfeeding. An evident time saver was a bottle machine that made the formula ready to drink. The mothers praised the machine for saving time and simplifying their lives: *“We were lucky to get the bottle machine as a birth gift. It is like a coffee machine for infants. It saved our lives; the nights became much more bearable.”* (M10).

Support from husbands was essential in getting through the day and very important to maintain breastfeeding, as it enabled the mothers to dedicate time to breastfeeding and to express milk:” *We were totally in it together. He [husband] always woke up with me and heated the bottles and arranged things. We were totally in it together; otherwise, the breastfeeding would never have worked.”* (M4) Most fathers were at home for the first two to three months. The situation could change drastically when the fathers returned to work:

*“It was around the time he [father] started to work. They were almost three months old. Then, it became difficult to be alone and pump and feed both. I fed them every three hours, and they did not sleep much or at least for very short periods during the days and nights. It became impossible to pump five or seven times a day and feed them [breast and bottle]. I lasted for about two or three weeks after he started to work. Then, I just quit [breast- and bottle feeding]”.* (M11)

#### Need for external support

The mothers felt that receiving help from grandparents was essential to making life manageable: *“We could not have done this without my parents. .. My mother sat by their [the twins] side while they slept so we could take a nap.”* (M10) Friends were also an invaluable resource for support. They and the grandparents helped with feedings, householding tasks, and taking care of siblings:

*“My friend who lives close by sometimes dropped by during lunchtime. .. She, of course, got coffee, but she also fed them [solids] and gave them a bottle [laughs]. .. It somehow turned into this: If someone comes, then he or she is handed the bottle.*” (M3)

A lack of relevant support and guidance from healthcare personnel was still present during months two to four; the LPT twins’ mothers repeatedly found that the primary healthcare nurses did not have sufficient knowledge and skills to guide them in sustaining breastfeeding. The mothers sought support on the internet and in twins’ mother’s groups on Facebook: *“I just Googled endlessly [for breastfeeding advice]. Google has sort of been my friend through this whole process.”* (M5).

## Discussion

This sequential explanatory mixed-method study aimed to describe the initiation and duration of any and exclusive breastfeeding for mothers of LPT twins and term twins during the first 4 months and to explore the breastfeeding experiences of mothers of LPT twins. This study is the first to describe the experiences of breastfeeding in mothers of LPT twins. We found that all LPT twins’ mothers initiated breastfeeding and that the rate up until 1 month was similar to that in mothers of term twins. However, more mothers of LPT twins than mothers of term twins ceased breastfeeding after the first month. The mothers of LPT twins found breastfeeding to be a complex and strenuous process where the key factors influencing the mothers’ experiences and decisions about breastfeeding were: the infants’ immature breastfeeding behaviors that required the mothers to use a breast pump alongside breastfeeding at the breast; the burden of following task-oriented feeding regimes; and the lack of guidance from healthcare professionals. Based on the mothers’ experiences and resources, they developed strategies to simplify the feeding process by ceasing breast milk expression or using only one feeding method per feeding. Throughout the process, support from fathers and grandparents was essential in sustaining breastfeeding. These results will be discussed in turn.

Iceland is a country with one of the highest breastfeeding rates in the world, with approximately 75% of infants breastfeeding at 6 months [35]. Thus, the norm to breastfeed is strong and subsequently influences mothers’ intentions to breastfeed, which most likely explains the high breastfeeding rate at 1 month. Previous studies have shown that prenatal intentions to breastfeed influence both the initiation and duration of breastfeeding for mothers of term and preterm infants [36, 37] and mothers of twins [11]. However, although the LPT twins’ mothers in our study intended and wanted to breastfeed and expected it to be hard work because they had two infants, they did not anticipate the effects of the infants’ prematurity. These effects are mainly related to hypotonia, immature state regulation, and ineffective sucking, which has been pointed out to be one of the main lactation risks associated with LPT infants [38–41] in combination with a delayed onset of lactation that is especially common among mothers of LPT infants [42, 43]. Therefore, it is recommended that mothers of LPT infants who intend to breastfeed express with a breast pump after each breastfeeding session or at least eight times in 24 hours to facilitate the phases of the lactogenesis process until breastfeeding is established [44, 45]. However, for the LPT twins’ mothers in our study, the burden of establishing breastfeeding at the breast with two LPT infants and striving to increase milk supply by using a breast pump after most feedings was complicated and difficult. Furthermore, the usage of multiple feeding methods and task-oriented feeding regimes was overwhelmingly complex and challenging and resulted in feelings of breastfeeding being instrumental and robotic. As a result, the mothers’ anxiety rose, and they became confused and exhausted, which affected their decisions on expressing breast milk and breastfeeding. Similar experiences of breastfeeding during the first weeks have been reported in studies with mothers of singleton late preterm infants [21, 46] and preterm infants [47]. After struggling for 1 month, the LPT twins’ mothers reached a limit / endpoint. They had put substantial effort into breastfeeding, but the desired outcome, exclusive breastfeeding, or breastfeeding without the use of a breast pump, was still uncertain, and the management of breastfeeding two infants was burdensome and complex. As a result, the mothers started to question the worth of their efforts, which led them to decide to change their management of breastfeeding. Radtke Demirci et al., [46], although including predominantly singleton LPT mothers, showed that the tension between uncertainty, motivation, and breastfeeding work seems to influence mothers’ decisions regarding breastfeeding.

Uncertainty is a well-known concept in the breastfeeding literature [48, 49]. Uncertainty underpins the model of “late preterm breastfeeding establishment” put forward by Radtke Demirci et al., [46]. The authors highlighted the multifaceted social, psychological, and biological nature of breastfeeding late preterm infants in combination with the unique circumstances among late preterm mother-infant dyads. The model suggests that a particular emphasis should be placed on contextual factors of breastfeeding LPT infants, such as the burden of having two infants, hospital practices, support from healthcare professionals and others, prior breastfeeding experiences, and social stressors. These factors correspond with the findings of our study.

There are several areas for possible interventions, which should ideally start in the hospital, and be followed up during the first months. Possible positive effects of a NICU stay on breastfeeding for singleton LPT infants have been reported previously [29, 46, 50] but have not been investigated for mothers of LPT twins. The positive effect is partially thought to be due to nurses and breastfeeding councilors who are accustomed to the breastfeeding challenges caused by prematurity and their expertise in assessing and supporting the establishment of an adequate milk supply, and in assisting in breastfeeding neurologically immature infants [51].

The support of fathers is very important in breastfeeding singletons [52, 53] and has been suggested to be even more important in breastfeeding twins [9]. However, there are no publications specifically on a father’s role in the initiation or duration of breastfeeding for twins. The support from fathers in our study was an essential part of getting through the day and was very influential in mothers managing and maintaining breastfeeding. Most of the mothers’ partners were on paternity leave for two to three months post-birth, which the mothers experienced as positive for their well-being and breastfeeding. Asking for and accepting help from important others is essential in getting through the day with twins [24], a notion that was often mentioned by the mothers in our study. Grandmothers are most often central figures in providing breastfeeding support to their daughters and daughters-in-law [54], and studies on grandparents wanting to be included and supportive during and after infant hospitalization in the NICU suggest their willingness and importance [55]. Therefore, in accordance with the model of “late preterm breastfeeding establishment” [46] and our findings, assisting mothers in identifying and seeking help from family and friends should be incorporated into the healthcare of LPT twins postpartum.

Many LPT twins’ mothers in our study reported lacking the skills and knowledge to manage breastfeeding. The knowledge and skills that midwives and primary healthcare nurses have in supporting and counseling mothers of LPT twins in breastfeeding and the clinical guidelines used in caring for mothers of LPT twins need to be critically examined. Previous research has noted that confusing or mismatching advice aimed at mothers of term infants is used for mothers of LPT infants from healthcare professionals where LPT infants and their mothers are not acknowledged as vulnerable and hence are not supported appropriately [40, 46, 56]. Additionally, mothers of twins often report that healthcare professionals give insufficient teaching specific to breastfeeding multiples, and they also receive conflicting advice about how to feed their infants [57, 58]. This suggests that the knowledge and skills to counsel, guide, and support breastfeeding mothers of twins and LPT infants must be reconsidered. Midwives and primary healthcare nurses should be educated on expected immature infant feeding behaviors; the importance of the early establishment of milk production with expressing, regardless of how breastfeeding seems to be progressing; ways to maintain milk production; how to progress from predetermined feeding schedules to cue-based feedings; and specific issues related to the breastfeeding of twins such as positioning and how to sustain breastfeeding in combination with bottle feeding. Additionally, preterm infants show more subtle breastfeeding cues, i.e., movements and signs, than term infants, and mothers of preterm infants require more guidance and support from healthcare professionals. We suggest that the way forward is to put emphasis on infant breastfeeding cues, where breastfeeding is valued for emotional aspects such as comfort and well-being [59], not for instrumental task-oriented activities where feeding regimes are prone to problems and failure.

### Strengths and limitations

The usage of a mixed-methods design is one of the strengths of this research, as the findings have been triangulated from different sources, which is important when exploring a complex phenomenon such as breastfeeding. Rigorously accounting for the literature related to the research topic, presenting and adhering to a protocol for the data analysis, and keeping track of the revisions made during the data analysis process increased the trustworthiness of the results [30]. We also report on the healthcare context of the study for the reader to further appraise the transferability of the results [30]. Furthermore, the composition of the research team can be seen as a strength for the research process, as HJ was not familiar with the area of breastfeeding, and RF was not familiar with the Icelandic context. An additional strength was that in both the quantitative and qualitative studies the total population was invited to participate. High response rates further enhances the validity of the findings.

A limitation is that the study samples were homogeneous; mothers were recruited from a single university hospital in Iceland, where breastfeeding is the norm and where most mothers were native, with high educational levels and income. Therefore, caution should be exercised when generalizing these findings to LPT twins’ mothers in more culturally and diverse socioeconomic settings. A limitation in the quantitative data is the lack of some important data, e.g., health characteristics, skin-to-skin contact, mothers’ support, and breastfeeding motivations. These are variables and experiences that could have shed further light on the underlying mechanisms. Our method of duplicating the answers of the few items of missing twin data could also be seen as a limitation. Self-report retrospective breastfeeding data are argued to be subject to social desirability and recall bias [60]. However, studies have found good validity of maternal recall for breastfeeding duration [61].

## Conclusions

We found that the initiation rate and duration of breastfeeding for mothers of LPT twins was similar to those of mothers of term twins until 1 month. However, significantly more mothers of LPT twins than mothers of term twins ceased breastfeeding after the first month. Our main explanations for this decline relate to the LPT mothers’ experiences of breastfeeding as a complex and strenuous process, where the key factors influencing their experiences and decisions about breastfeeding were: infants’ immature breastfeeding behaviors that required mothers to use a breast pump alongside breastfeeding; the burden of following task-oriented feeding regimes; and the lack of guidance from healthcare professionals. As a result, the mothers started to question the worth of their breastfeeding efforts, which led them to change their management of breastfeeding with diverse outcomes during the following months. Support from fathers and grandparents was essential throughout the process of sustaining breastfeeding. The study findings highlight that postpartum and primary care for this vulnerable group should be provided by highly qualified healthcare professionals who are knowledgable of the impact of late prematurity and twins on the progression of breastfeeding and differentiated from routine postpartum and primary care. This study also highlights the need for developing specific interventions and policies aimed at LPT twins and their mothers.

## Supplementary Information

**Additional file 1: Supplemental Table 1.** Background of services to mothers and infants.

## Data Availability

The data that support the findings of this study are not openly available due to patient confidentiality concerns but are available from the corresponding author upon reasonable request.

## Abbreviations

EPDS: Edinburgh Postnatal Depression Scale
LPT: Late preterm
NICU: Neonatal intensive care unit
NUH: The National University Hospital of Iceland
SD: Standard deviation
