# Supplementary material for: Breastfeeding initiation, duration, and experiences of mothers of late preterm twins: a mixed-methods study
Source: Int Breastfeed J. 2022 Sep 8;17:68. doi: 10.1186/s13006-022-00507-3 (PMC9461222; doi:10.1186/s13006-022-00507-3)
Supplement: Supplementary file 1 — Additional file 1: Supplemental Table 1. Background of services to mothers and infants. [file 13006_2022_507_MOESM1_ESM.docx]

Supplemental Table 1. Background of services to mothers and infants

| **Service/Setting/Management** | **Description/Role** |
| --- | --- |
| Health care system | The health care system in Iceland is universal and public-funded. Midwifery service, breastfeeding consultant service, and well-baby follow-up at home or at the community health centers are paid by the Icelandic health insurance system. |
| Birth service | The National University Hospital of Iceland (NUH) is the largest birth facility and the main referral hospital for high-risk pregnancies and childbirths in Iceland, with 70 to 75% of all deliveries and about 95% of multiple births. In addition, the only Neonatal Intensive Care Unit (NICU) in Iceland is located at the NUH. |
| Maternity Unit | Infants born ≥35 gestational weeks without medical problems are cared for at the maternity unit of NUH, where they room‐in with the mother and father. The official policy of the maternity unit is that most primiparas are to go home within 24 hours and multiparas within 12 hours from delivery and mothers with twins before 72 hours^[[1]](#footnote-1)^. Mothers that have had a cesarean section can stay up to 48 hours. The midwife at the maternity unit contacts a home midwife that has a contract with the Icelandic health insurance before the mother and infants are discharged to set up a home service. |
| Neonatal Intensive Care Unit | A 14‐bed, level IIIb unit. All preterm infants under the gestational age of 35 weeks and with birth weight less than 2,000 g are admitted to the NICU, along with newborn infants with gestational age more than 35 weeks with medical complications. The NICU is open to parents 24 h a day, and every infant’s cot or incubator has chairs for parents. However, there are limited resources for mothers to room‐in with their infants. During the quantitative study, the unit had two rooms for rooming‐in. However, during the qualitative research, there were five rooms for rooming-in.  Infants are discharged home from the NICU when an infant has reached 35 weeks gestation, with no apneas and without apnea medication for at least a week, and vital signs within normal range. Many preterm infants need a feeding tube at this time. There is an early-discharge program that experienced neonatal nurses lead within the NICU. Parents are prepared for going home with an infant mainly feeding through a feeding tube, with education about the feeding tube, first aid, and breastfeeding/feeding. After going home parents have access to nursing support through 24 hours telephone contact and bring the infant to the unit for a checkup and advice from the nurse every 3-5 days until the feeding tube is out. |
| Home service from midwives and breastfeeding consultants, contract with Icelandic health insurance. | Midwifes can have a contract with the Icelandic health insurance, and those that have can, through that contract, service mothers after birth at the mother's home. This service is free for the mother, and the midwife gets paid through Icelandic health insurance. To be abel to get that service, the mother must leave the hospital with her children within 72 hours from birth^[[2]](#footnote-2)^.  Healthy mothers delivering singleton infant going home with their infant within 4 or 48 h of delivery receive five to seven home visits from a midwife during the first ten days after birth. Mothers with twins and mothers that are too sick and/or have an infant that is too sick to go home within 48 hours, can get an exemption/permission from a midwife and/or neonatologist to go home within 72 hours and still get seven home visits. If mother and/or infant/s leave the hospital after 72 hours from birth, they don't get a midwife service at home.  If the home visiting midwife assesses that the mother has severe breastfeeding problems, she can consult a breastfeeding consultant to visit the mother, free of charge, for two visits. Once before the infants are ten days old and once before 14 days. The breastfeeding consultants get paid through Icelandic health insurance for those two visits. |
| Well-baby clinics/ primary health care/community health centers. | For all infants, the primary health care nurse visits the family two to three times from about seven to 10 days after coming home until infants are six to nine weeks old. After this time, parents bring their infants to the community health clinics to meet a primary health care nurse or a physician for well-baby checkups and immunizations. This service is free of charge for all parents. |
| Parental leave | At the time of the first study, the paid parental leave in Iceland for parents of twins was a total of 12 months, six months for the mother, three months for the father, and three months that parents could decide on. The qualitative study happened when the government was increasing the parental leave, from June to 31. of December 2020, mothers got four months and fathers four months, and parents could decide on five months, a total of 13 months. From January 2021, each parent got six months plus three months they could share between them, a total of 15 months. The exact numbers on how many use the parental leave and for how long time are not available for the research period. It is estimated that over 90% of mothers use all their rights and about 80% of fathers use some. In 2017 of the fathers that used their rights for parental leave the average time used was 30% of their total right^[[3]](#footnote-3),^^[[4]](#footnote-4)^. The rights for a paid parental leave for parents of twins during the research period was from 12 to 15 months^[[5]](#footnote-5)^. |
| National breastfeeding recommendations | The Icelandic Directorate of Health recommends that all infants are exclusively breastfed until six months of age, with some degree of breastfeeding until they reach the age of 1 year. It is also recommended that infants start solids at six months unless the breastfed infant is not growing appropriately. Then, the advice is to start solids and/or formula at 4-6 months of age^[[6]](#footnote-6)^ . |

1. [↑](#footnote-ref-1)
2. 1 <https://www.landspitali.is/sjuklingar-adstandendur/deildir-og-thjonusta/medgongu-og-saengurlegudeild/>

   <https://www.ljosmaedrafelag.is/thjonusta/heimathjonusta/log-og-samningar-og-leidbeiningar> [↑](#footnote-ref-2)
3. <https://www.althingi.is/altext/pdf/150/s/0241.pdf> [↑](#footnote-ref-3)
4. Johannsdottir, K. (2018). Fæðingarorlof feðra. Orlofsnýting og áhrif. [Lokaverkefni til MA-gráðu í félagsráðgjöf til starfsréttinda. Háskóli Íslands]. <https://skemman.is/bitstream/1946/31986/1/MAritgerð-Fæðingarorlof%20feðra-KarlottaJóhannsdóttir.pdf> [↑](#footnote-ref-4)
5. <https://www.vinnumalastofnun.is/en/maternitypaternity-leave-fund/first-steps> [↑](#footnote-ref-5)
6. <https://www.landlaeknir.is/servlet/file/store93/item30469/> [↑](#footnote-ref-6)
